# Light sintering of ultra-smooth and robust silver nanowire networks embedded in poly(vinyl-butyral) for flexible OLED

**DOI:** 10.1038/s41598-018-32590-0

**Published:** 2018-09-21

**Authors:** Dong Jun Lee, Youngsu Oh, Jae-Min Hong, Young Wook Park, Byeong-Kwon Ju

**Affiliations:** 1 Display and Nanosystem Laboratory, College of Engineering, Korea University, Seoul, 136–713 Republic of Korea; 2 Photo-Electronic Hybrids Research Center, Korea Institute of Science and Technology (KIST), Seoul, 02792 Republic of Korea; 3 Institute of Advanced Composite Materials, Korea Institute of Science and Technology (KIST), Jeonbuk, 55324 Republic of Korea; 4 School of Mechanical and ICT Convergence Engineering, SUN MOON University, Chungcheongnam-do, 31460 Republic of Korea

## Abstract

A conductive, uniform, and ultra-smooth flexible transparent composite film is produced by embedding silver nanowires (AgNWs) into poly(vinyl-butyral) (PVB) without pressure or high-temperature annealing. The adhesion of AgNWs was greatly improved by embedding them in PVB, and surface roughness and sheet resistance (R_s_) improvements were achieved through the use of the intense pulsed light (IPL) method, which welds the interconnections among AgNWs in a short time without heat or pressure treatment. The sheet resistance of PVB/AgNWs with the IPL(PAI) composite film reaches 12.6 ohm/sq with a transmittance of 85.7% (at 550 nm); no clear changes in the sheet resistance are observed after a substrate bending and tape test, suggesting excellent flexibility. In the case of PAI, the change in sheet resistance was only 2.6% after a 2,000-bend test, and the resulting bending radius was less than 1 mm. When IPL was exposed to PVB/AgNWs, the figure of merit was 2.36 times higher than that without exposure. Finally, flexible OLEDs using PAI exhibited comparable or higher electroluminescent characteristics than other devices with well-known flexible electrodes—including indium-zinc-oxide on polymer plastic—which is a promising discovery for flexible optoelectronic applications.

## Introduction

With the increasing popularity of smartphones and tablets, the demand for flexible, low-cost, and transparent conductive films instead of expensive and brittle sputtered indium tin oxide (ITO) films has increased. This has prompted and stimulated the development of transparent conductive materials. To obtain a transmittance of 90% in the visible spectrum and a sheet resistance under 20 ohm/sq, it is necessary to heat the substrate at a high temperature (300–500 °C) to achieve better crystallization of the metal oxide^1–3^. However, the high temperature process, which is the production method for ITO, is not suitable for the polymer substrates needed in flexible devices. These problems have motivated research on alternative transparent electrodes to ITO.

As candidates, various alternatives have been investigated: silver nanowires (AgNWs), conducting polymers^4,5^, metal grids^6,7^, and carbon materials such as graphene^8^, carbon nanotubes^9,10^ reduced graphene oxide^11^, etc. Among these candidates, solution-treated carbon materials have been studied extensively because of their easy fabrication and low cost. However, their sheet resistance is quite high (100 to 2000 ohm/sq) and their transmittance of 80–86% is lower than that of ITO^8–11^. Thus, original carbon-based materials do not meet the requirements for optical devices.

On the other hand, AgNW thin films, which are excellent in transparency and sheet resistance, have been proposed as a cost effective and sustainable alternative to ITO^12–18^. Moreover, using AgNWs is advantageous because they can be prepared by low-cost processes such as spin-coating, drop-casting, rod-coating, and spray-coating. However, AgNWs also have some drawbacks when used as optoelectronic substrates.

The main difficulties for the application of AgNW thin-films as transparent electrodes are intrinsic roughness, weak adhesion, and high contact resistance to the substrate^19,20^.

Electrical conduction in an AgNWs network is affected by the resistances at the junctions of AgNWs, because of the percolative nature of conduction. Methods such as thermal annealing and mechanical pressing have been proposed to reduce junction resistances among AgNWs; however, these methods have some drawbacks that include the limited availability of substrates that can withstand high temperatures and the need for complex and expensive equipment^12,21^.

In contrast to the conventional pressure and thermal sintering technique in which the sample is exposed continuously to high temperature, the intense pulsed light (IPL) photonic sintering technique irradiates a sample with multiple short flashes, each with a pulse length in the range of a few microseconds to a few milliseconds^21–27^. Photonic sintering is used mainly on metal films that have a higher absorbance of radiant lamp energy, which results in a faster rise of temperature of the metal film. Substrate damage is prevented by the very short exposure to light^21^.

Previously, many studies have sintered AgNW using IPL exposure, high-intensity pulsed light (HIPL)^28^, by coupling with graphene^29^, combining UV-C irradiation and white flash light^30^, applying AgNW to a stretchable substrate^31^, femtosecond exposure^32^, and via an analysis for IPL exposure^33^. However, papers on OLED devices are rare because complex technology is required to fabricate OLEDs.

Another limitation is that the AgNWs film has been found to be unsuitable for many thin-film optoelectronic devices owing to its inherent roughness and low adhesion. The high peaks produced by overlapping junctions between the wires further increase the surface roughness.

The weak adhesion of AgNWs reduces the stability of a device’s operation. To solve these problems, AgNWs were embedded in poly(vinyl-butyral) (PVB) to reduce roughness and increase adhesion to a substrate. The PVB used is a low-cost alternative that offers strong binding capability, flexibility, optical clarity, and adheres to many surfaces^34^.

In this study, we demonstrate a scalable and simple solution processing method to fabricate improved conductive, flexible, and ultra-smooth PVB/AgNWs with an IPL (PAI) composite electrode without high pressure or temperature treatment. The AgNWs networks are completely welded and suitably embedded into the PVB layer by light-induced heat transfer, which increases the stability and electrical conductivity of the entire film. The PAI composite film has a uniform and smooth surface suitable for the fabrication of thin-film optoelectronic devices. The flexible organic light-emitting diodes (OLEDs) with the PAI composite electrode show enhanced electroluminescent characteristics compared to a device fabricated on well-known flexible poly(ethylene terephthalate) (PET)/indium zinc oxide (IZO) electrode.

## Results and Discussion

### Fabrication of the transparent electrode

**Figure 1:**
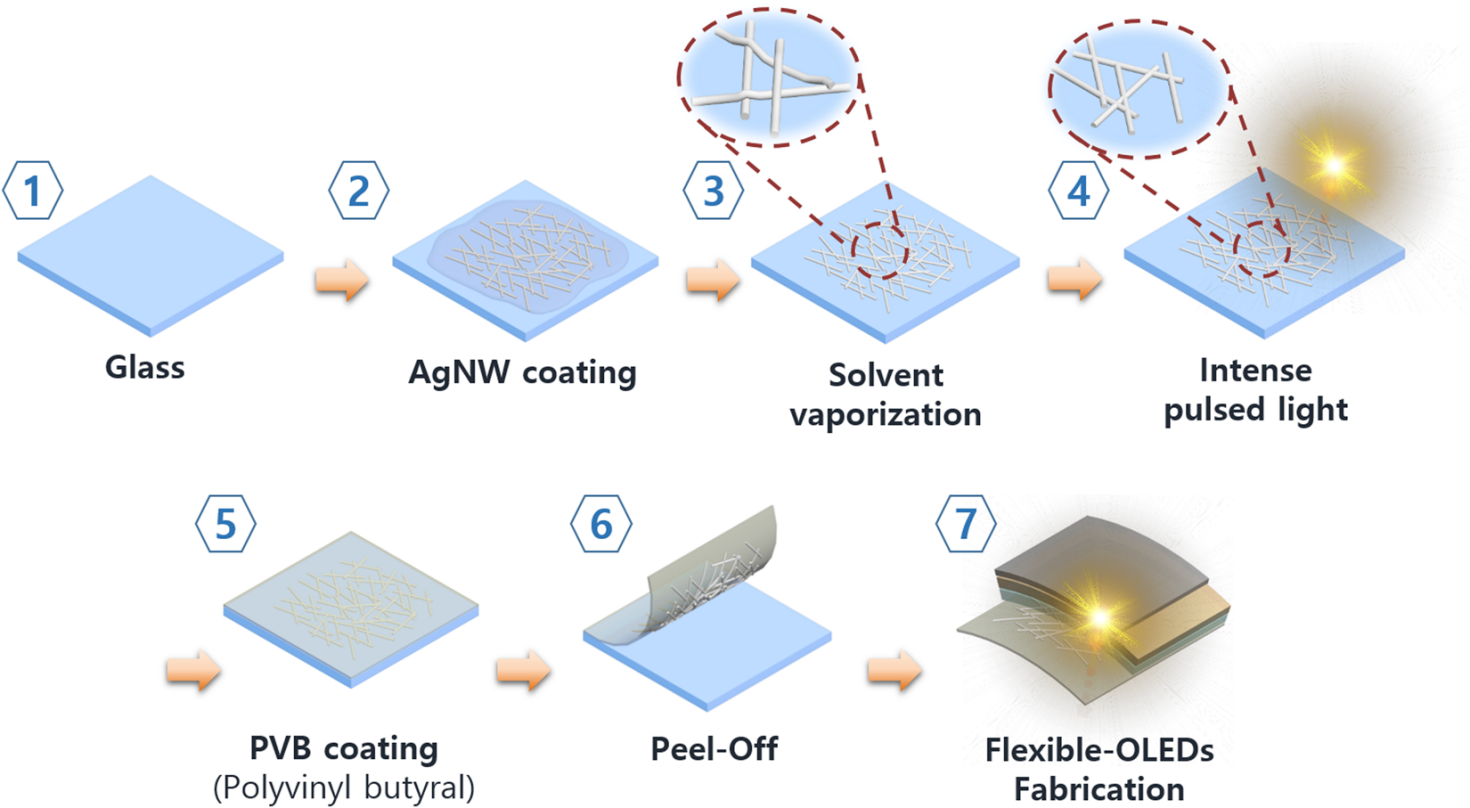
Schematic of PVB/AgNWs composite transparent electrode fabrication process.

The procedure used for the fabrication of the AgNWs-based transparent electrode is schematically illustrated in Fig. 1. The glass substrates (Eagle XG, Corning Co., Ltd.) were sequentially cleaned in acetone, methanol, and deionized water by ultra-sonication for 10 min each, followed by drying with nitrogen gas.

The dispersion of AgNWs was spin-coated (500 rpm for 20 s) onto the glass and heated on a hot plate at 60 °C for 5 min to remove any remaining organic solvent.

After de-hydration, the samples were exposed to 500 μs pulses of intense light using a photonic sintering system operating at an input voltage of 200 V (4 J/cm^2^) to form the welded interconnections^35^ of AgNWs to not only reduce the sheet resistance, but also to improve the adhesion between the AgNWs and PVB by reducing surface roughness.

To fabricate the flexible substrate, the polymer solution was prepared as follows; 2 g of PVB was dissolved in N,N′-dimethylformamide (DMF, 20 mL) by sonication for 5 min, and 0.3 g of hexamethylene diisocyanate (HDI) was added into the solution. PVB was spin-coated (300 rpm for 20s) on the silver-nanowire films. The samples were crosslinked few hours at 70 °C in an oven. Once the PVB film was formed on the AgNWs electrodes, the sample was soaked in water for 10 min to induce hygroscopic swelling of the PVB film to allow the film to be safely peeled off from the preliminary glass substrate.

A Poly(3,4-ethylenedioxythiophene)-poly(styrenesulfonate) (PEDOT:PSS) solution with a binding system (Clevios PH 1000, Heraeus Co., Ltd.) was then spin-coated at 2000 rpm onto the AgNWs network to planarize its surface followed by dehydration on a hot-plate.

The reference oxide transparent electrodes were fabricated as follows. For the reference, the well-known flexible transparent conductive IZO oxide electrodes (200 nm) were deposited onto the poly(ethylene terephthalate) (PET) substrate, and amorphous 130 nm thick indium tin oxide (ITO) coated PET substrates were purchased from Sigma-Aldrich.

### Surface properties of AgNWs embedded flexible transparent electrode

**Figure 2:**
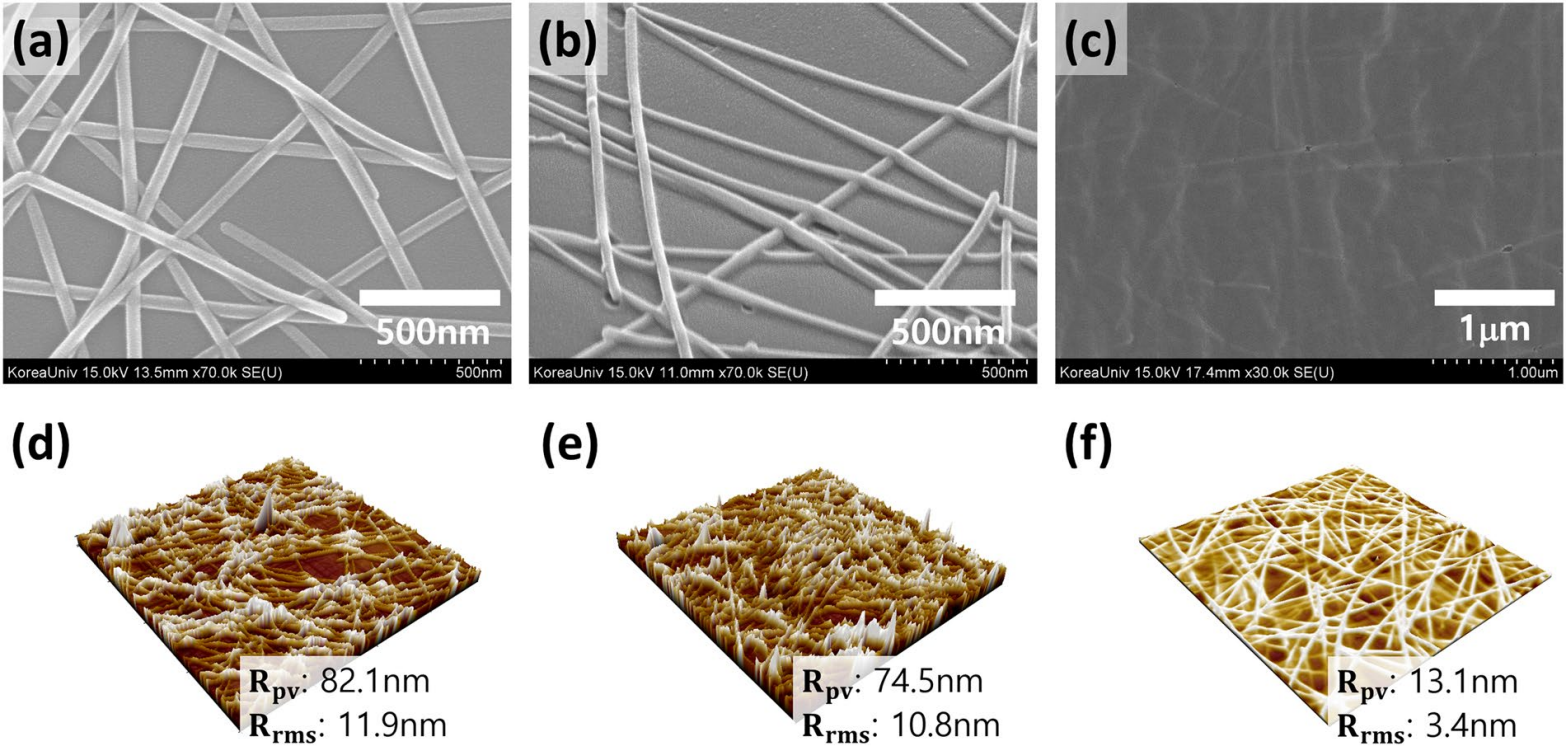
SEM images of (**a**) Glass/AgNWs, (**b**) Glass/AgNWs with IPL, and (**c**) PVB/AgNWs. AFM topography images of (**d**) Glass/AgNWs, (**e**) Glass/AgNWs with IPL, and (**f**) PVB/AgNWs.

To confirm that the silver nanowire electrodes were properly embedded or adhered to the surface of the PVB film, we investigated the surfaces of the film using a scanning electron microscope (SEM) and atomic force microscopy (AFM). Figure 2 shows the SEM and AFM images of the AgNWs film on glass substrate (Fig. 2a,d), AgNWs with IPL process on glass substrate (Fig. 2b,e), and AgNWs embedded in PVB with IPL electrode (Fig. 2c,f).

The surface roughness of the spin-coated AgNWs layer on the rigid substrate was significantly higher than the commercially available ITO films. A root-mean-square surface roughness (R_rms_) of 11.9 nm and maximum peak-to-valley (R_pv_) range of 82.1 nm were measured for the AgNWs layer. The presence of protruding nanowires, which induces the high R_pv_, leads to localized height elevations and this large surface fluctuation induces a higher possibility of electrical shorts in the devices. It is obvious that, apart from transparency and conductivity, surface roughness is clearly another attribute that affects the compatibility of transparent electrodes with the device.

In contrast, Fig. 2(b) shows AgNWs welded together at each contact point by the IPL process. The IPL process forms the AgNWs connections and reduces sheet resistance. The surface roughness is also slightly reduced.

The AgNWs embedded in PVB electrode displays an ultra-smooth surface with an RMS of 3.4 nm and R_pv_ of 13.1 nm, as can be seen from the high magnification AFM images in Fig. 2(f). This ultra-smooth surface morphology, which is better than AgNWs coated on glass, indicates that the spin-coated PVB liquid polymer well permeated the AgNWs network, filled the holes in the network, and filled the voids at the interface between AgNWs and the rigid substrate.

### Sheet resistance and Transmittance analysis

**Figure 3:**
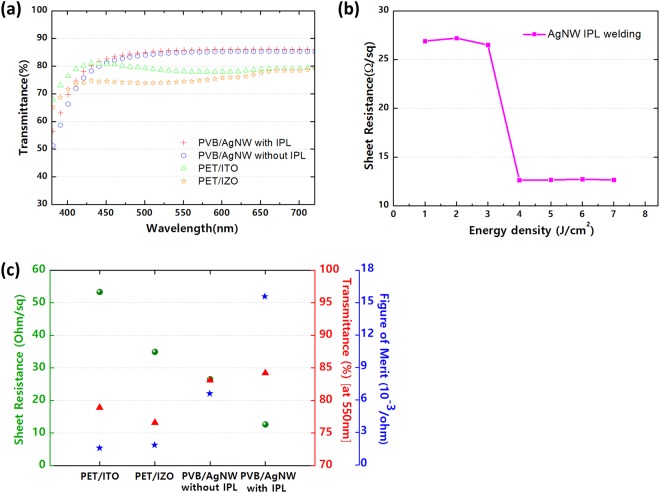
(**a**) Transmittance spectrum of IPL-treated and non-treated pristine PVB/AgNWs, PET/ITO, and PET/IZO films. (**b**) Sheet resistance response to the intense pulsed light method induced welding. (**c**) Figure of merit values for relative sheet resistance and transmittance values of the PET/ITO, PET/IZO, and PVB/AgNWs films with and without IPL.

Figure 3(a) shows the optical transmittance characteristics. Both PVB/AgNWs electrodes exhibited a high optical transmittance in the visible region, which was comparable to the PET oxide samples. The optical transmittance at a wavelength of 550 nm and the average transmittance values between 400 nm and 700 nm are summarized in Table 1. Owing to the thinness of the PVB and the open space of the AgNWs, both PVB/AgNWs electrodes exhibited high optical transmittance, which are acceptable in the fabrication of OLEDs. Fig. 3(a) shows the effect of using IPL exposure on the optical transmittance of the PVB/AgNWs structure, revealing that although the change in transmittance is negligible, haziness increased over a wide range of visible wavelengths and the total transmittance increased slightly^36^.

**Table 1:** Summary of sheet resistance, transmittance at 550 nm, average transmittance at 400–700 nm, and figure of merit of each type of conductive film.

Condition	PVB/AgNWs with IPL	PVB/AgNWs without IPL	PET/ITO	PET/IZO
Sheet resistance [Ohm/sq]	12.6	26.5	34.9	53.3
Transmittance at 550 nm [%]	85.7	84.9	74.3	78.1
Average transmittance (400–700 nm [%])	84.2	83.1	76.6	78.9
Figure of Merit (FoM)	15.6	6.6	1.8	1.6

In Fig. 3(b), the sheet resistance is expressed as a comparison to varying IPL energy densities. When the IPL interval was held constant at 500 μs, irradiation energies greater than 4 J/cm^2^ considerably reduced the AgNWs sheet resistance in every instance. This provides support for similar, and even improved, property enhancements for AgNWs when compared against longer time-consuming furnace heat treatment and high pressure processes. Additional improvements to the AgNWs sheet resistances were not seen at energies higher than 4 J/cm^2^. This is attributed to the welds which exist in a more binary state. In other words, once the welds are established at a certain threshold energy, any additional energy supplied to the system does not increase the degree to which the AgNWs are welded together along the AgNWs network.

Figure 3(c) and Table 1, show the electrical and optical properties of the PVB/AgNWs composite films and reference PET-based conductive films.

Compared to the PET/IZO reference sample, the PVB/AgNWs films exhibited much lower sheet resistance of 12.6 Ω/square (with IPL) and 26.5 Ω/square (without IPL), respectively. The PVB/AgNWs yielded a lower sheet resistance than typical PET/IZO owing to the better connectivity of the Ag network.

The sheet resistance (R_s_) and transmittance (T) of the PVB/AgNWs films are closely related to the AgNWs density and PVB thickness. R_s_ and T values at 550 nm were used to calculate the figure of merit (FoM, Φ_TC_) as defined by the Haacke equation^37^.

1 $${{\rm{\Phi }}}_{{\rm{TC}}}=\frac{{{\rm{T}}}^{10}}{{{\rm{R}}}_{{\rm{S}}}}$$

Figure 3(b) depicts the R_s_, T, and calculated Φ_TC_.

The electrical and optical characteristics of the IPL-exposed PVB/AgNWs films are much improved compared with the unexposed PVB/AgNWs films owing to sintering of the AgNWs through IPL exposure. The haze was increased by the scattering effect at the welded-AgNWs junctions, and as a consequence, the total transmittance was also increased^30^ (Supporting Information, Fig. S2). Therefore, AgNWs with IPL has the highest FoM because of its low sheet resistance and high transmittance.

### Extreme deformation of AgNWs embedded flexible transparent electrode

In addition to the excellent electrical and optical properties, the PAI samples possess superior mechanical flexibility, which is essential for emerging optoelectronic devices such as flexible displays.

**Figure 4:**
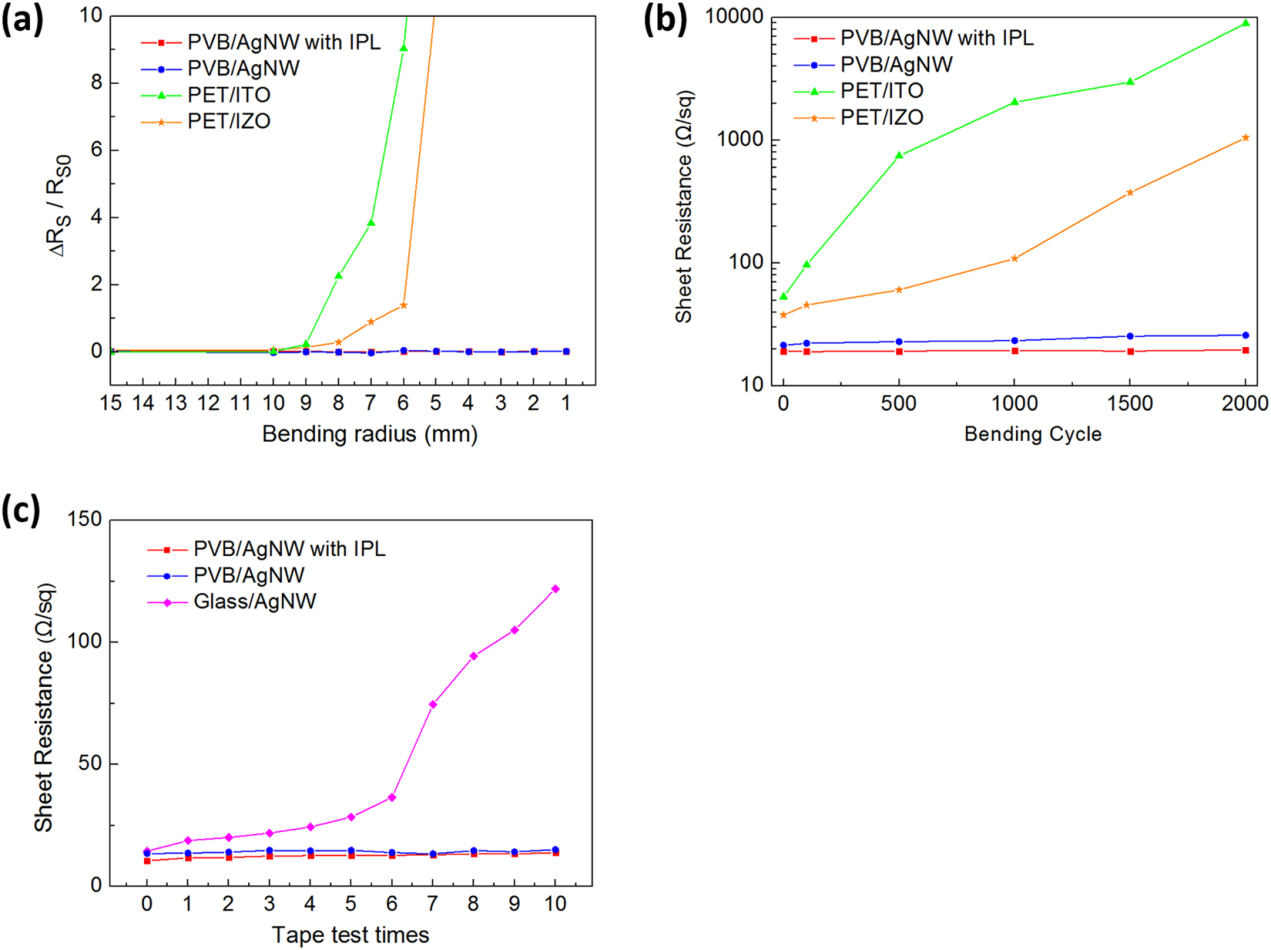
(**a**) Measured normalized sheet resistance variation (ΔR_s_/R_s0_ = (R_s_(bent) − R_s0_(unbent))/R_s0_(unbent)) of PVB/AgNWs, ITO (t_ITO_ = 130 nm), and IZO (t_IZO_ = 200 nm) electrodes on a PET substrate (t_PET_ = 200 µm) as a function of bending radius. (**b**) Sheet resistance change of the PVB/AgNWs samples with and without IPL exposure, PET/IZO, and PET/ITO electrodes as a function of number of bending cycles. (**c**) Sheet resistance change of the PVB/AgNWs and Glass/AgNWs electrodes as a function of number of tape tests.

We conducted bending radius (R_b_) tests on the PVB/AgNWs composite films, with results shown in Fig. 4(a) and Table 2. PVB/AgNWs composite films and ITO or IZO films on PET were prepared for comparison in bending radius tests. PVB/AgNWs composite films maintained almost constant R_s_ as the bending radius decreased from 15 mm to 1 mm. In contrast, for the ITO and IZO films, R_s_ increased dramatically at R_b_ ≈ 9 mm and could not be measured for R_b_ ≈ 7 mm.

**Table 2:** Summary of bending characteristics of conductive films.

Condition	PVB/AgNWs with IPL	PVB/AgNWs without IPL	PET/ITO	PET/IZO
Ultimate bending radius	<1 mm	<1 mm	≈7 mm	≈9 mm
R_s_ change rate (after 2000 times)	+2.6%	+20.4%	+2677.8%	+166691.7%

Figure 4(b) compares the mechanical flexibility of the PAI, pristine PVB/AgNWs, PET/IZO, and PET/ITO films as a function of bending cycles. As shown in Fig. 4(b), all samples are rolled around a bending radius (R_b_) of 10 mm, subsequently unrolled at a speed of 40 mm/s, and the sheet resistance of each transparent conductive film is compared to its initial value.

As shown in Fig. 4(b), within the first few cycles of this process, the PET/ITO film begins to crack, resulting in a sharp increase in sheet resistance. ITO is inherently brittle and cracks when exposed to a minimal amount of strain. However, for the PVB/AgNWs composite films, the embedded-structure is mechanically flexible owing to its asymmetrical and random shape. Therefore, the sheet resistance of the PVB/AgNWs composite films remains unchanged after more than 2,000 bending cycles.

In Table 2, the rate of change of sheet resistance (ΔR_s_) after 2,000 bending times in PET/ITO was almost infinite. In contrast, there is almost no change in PAI compared to PET/IZO and PET/ITO, and the rate of sheet resistance change is only 2.6% for samples exposed to IPL.

The PVB/AgNWs composite film was also subjected to an adhesion test using 3M Scotch tape. Figure 4(c) shows the graph of PVB/AgNWs composite films and Glass/AgNWs transparent conducting electrodes (TCE) after peeling the film repeatedly with the tape. The adhesion test, with repeated taping and releasing, revealed that the AgNWs embedded in the PVB were very robustly adhered to the PVB, as shown in Fig. 4(c). The graph clearly shows that AgNWs was easily fully separated due to its loose adhesion to the glass substrate while the PVB/AgNWs composite films were only slightly separated.

The rate of change of each sheet resistance according to the tape test was evaluated, and the largest rate of change for the three types of samples is shown in Table 3. The sheet resistance value for each tape test is represented by R_s n_, and R_s n−1_ represents the previous sheet resistance value. The formula used is as follows.

2 $${\rm{Largest}}\,{\rm{rate}}\,{\rm{of}}\,{\rm{change}}\,[ \% ]=({{\rm{R}}}_{{\rm{s}}{\rm{n}}}-{{\rm{R}}}_{{\rm{s}}{\rm{n}}-1})/{{\rm{R}}}_{{\rm{s}}{\rm{n}}-1}\times 100$$

**Table 3:** The largest aspect resistance change rate during the tape test of fabricated TCEs.

Condition	Glass/AgNWs	PVB/AgNWs without IPL	PVB/AgNWs with IPL
The largest rate of change	260%	11%	9%

For Glass/AgNWs, the highest rate of change in sheet resistance occurred when the tape test was conducted seven times, with an increase of approximately 260%. On the other hand, PVB/AgNWs without IPL shows the second largest rate of sheet resistance change of 11% (after one tape test). This is because the AgNWs is embedded into the PVB, which increases its adhesion. In the case of PAI, the largest rate of change of the sheet resistance was 9% after eight tape tests. The higher adhesion of PAI than PVB/AgNWs without IPL results from the welding effect caused by IPL exposure. Thus, the mechanical stability of PVB/AgNWs with IPL exposure is expected to be suitable for applications in flexible optoelectronic device.

### Comparison of other transparent electrodes with PVB/AgNWs electrodes through OLED characteristics

**Figure 5:**
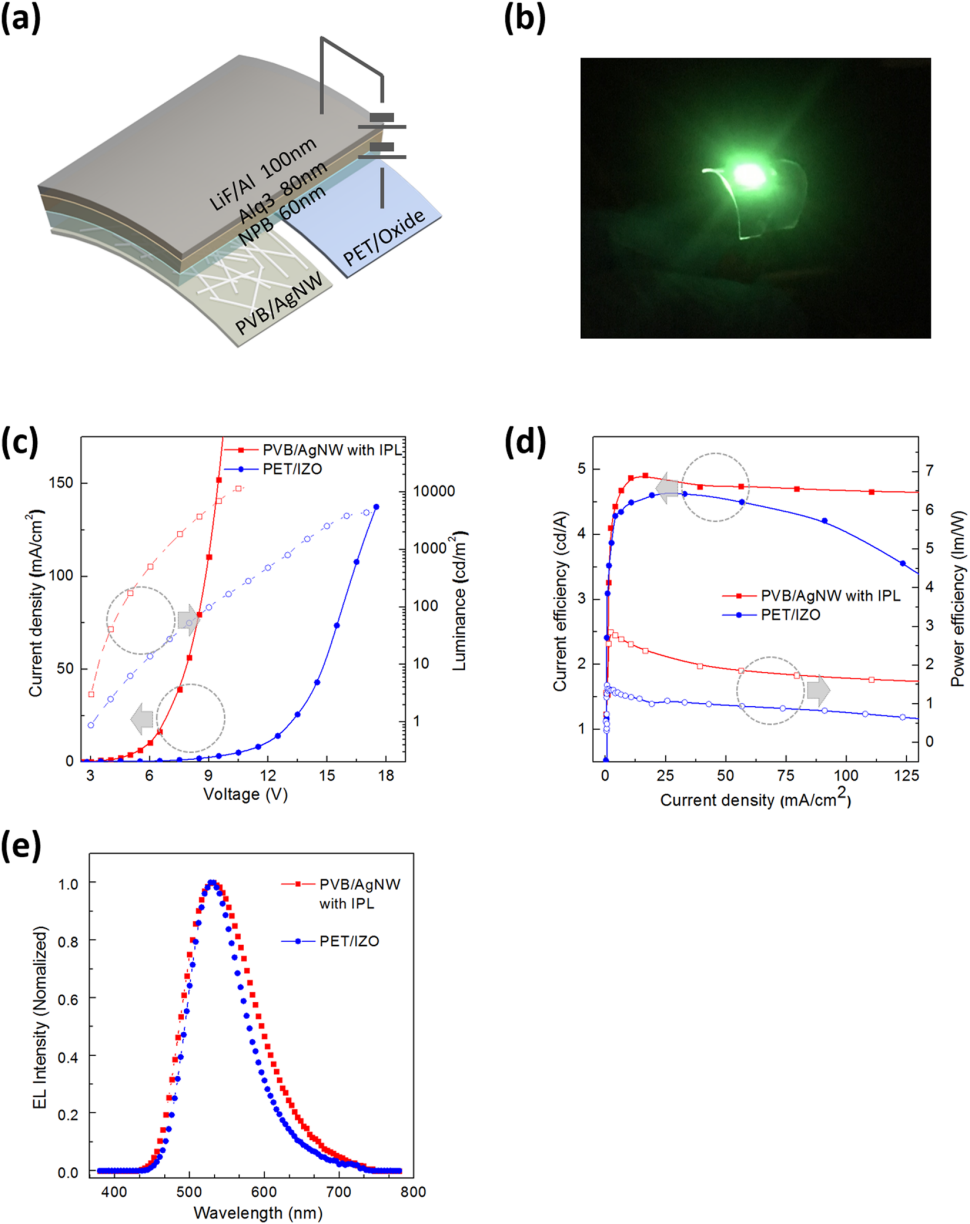
Characteristics of OLED devices with PVB/AgNWs with IPL composite and IZO anode. (**a**) Schematic section of the OLED device. (**b**) Photographic images of operating PVB/AgNWs-based OLEDs in flexible modes, compressive stress. (**c**) Current density–voltage-luminance characteristics. (**d**) Current efficiency and power efficiency as a function of luminance. (**e**) Normalized EL intensity versus wavelength.

The transparent and flexible embedded AgNWs fabricated using spin-coating, IPL exposure, and transfer method were successfully used in OLEDs, as shown in Fig. 5(a). The basic structure of our OLED consisted of six layers: a PAI anode, a PEDOT:PSS hole-injecting layer, a N,N′-Bis(naphthalen-1-yl)- N,N′ -bis(phenyl)-benzidine (NPB) hole transport layer, a tris(8-hydroxyquinolinato) aluminum (Alq_3_) emitting layer, an lithium fluoride (LiF) electron-injecting layer, and an Al cathode layer. With the exclusion of PEDOT:PSS, all materials required for the fabrication of this device were thermally evaporated sequentially.

Figure 5(b) shows photographs of the devices when bending. This clearly demonstrates that the PAI electrode is suitable as a TCE in flexible organic electronics owing to its excellent electrical, optical, and mechanical properties.

Figure 5(c–e) show the plots of the performance of the OLEDs based on the PAI in comparison with the performances obtained using PET/IZO electrodes, where IZO was used as the flexible electrode.

Figure 5(c) shows the current density-luminance-voltage characteristics of the OLEDs prepared using PAI or IZO. The device fabricated with PET/IZO displayed a higher operating voltage, lower current density, and lower luminance (1.14 mA/cm^2^ and 52.55 cd/m^2^, respectively, at 8 V) than the device fabricated with PAI (56.28 mA/cm^2^ and 2670 cd/m^2^, respectively, at 8 V). The higher sheet resistance of PET/IZO (35 Ω/sq, 74% transmittance at 550 nm) than PAI (13 Ω/sq, 85% transmittance at 550 nm) impeded the spread of a uniform current across the entire anode area and hampered the balanced recombination of electrons and holes in the device. The PAI devices exhibited the lowest leakage current below the light emission threshold voltage of 3 V.

The current efficiency (CE) and power efficiency (PE) of the devices are shown in Fig. 5(d) as a function of the luminance, and Table 4 summarizes this comparison.

**Table 4:** Device characteristics of OLEDs on PVB/AgNWs with IPL electrodes fabricated in this study and PET/IZO.

Device	Maximum CE^a^ [cd/A]	Maximum PE^b^ [lm/W]	Average^c^		Standard Deviation^c^	
			CE [cd/A]	PE [lm/W]	CE [cd/A]	PE [lm/W]
PVB/AgNWs with IPL	4.91	2.86	4.70	1.75	0.04	0.18
PET/IZO	4.68	1.48	4.11	0.83	0.52	0.16

^a^Maximum CE: maximum current efficiency. ^b^Maximum PE: maximum power efficiency. ^c^Current density from 25 mA/cm^2^ to 150 mA/cm^2^.

Table 4 indicates that the PVB/AgNWs devices achieved a maximum CE of ≈4.91 cd/A, which was higher than PET/IZO (CE ≈ 4.68 cd/A). The average CE value of PVB/AgNW is 4.70 cd/A and the average value of PE is 1.75 cd/A. This is 14.3% higher than the average CE value of PET/IZO, and the average PE value is 111% higher. The standard deviation of PVB/AgNW with IPL is very small, and is less than 0.2 for both CE and PE. On the other hand, the standard deviation of CE for PET/IZO is 0.52 cd/A, which indicates a relatively large change, as shown in Fig. 5(d).

Figure 5(e) plots the EL spectra of OLED devices with PAI and IZO film on PET as anodes which were measured at 500 cd/m^2^. Both types of devices show almost the same spectrum, which has a peak at ≈530 nm. This indicates that there is no color distortion even when using the PAI electrode. PET/IZO has a narrower full width at half maximum (FWHM) than PVB/AgNW with IPL. This is because the micro-cavity phenomenon^38^ occurs owing to the higher reflectivity of PET/IZO (Supporting Information, Fig. S3).

Overall, the PAI prepared using our method represents a good strategy for fabricating portable optical instruments, flexible displays, and lighting applications, because the embedded and welded AgNWs effectively replaces conventional brittle transparent oxide electrodes while providing comparable current efficiencies.

In conclusion, we demonstrated that a flexible transparent conducting AgNWs film with superior mechanical characteristics and low surface roughness can be fabricated by embedding an AgNWs film at the surface of a transparent PVB. The conductivity properties of AgNWs networks can be improved by using the radiation produced by an IPL. The resistance change rate of the banding test characteristics also decreased by 17.8% after IPL exposure. Materials preparation involves solutions at atmospheric pressure requiring short processing time. Through this simple method, various flexible and transparent devices can potentially be fabricated, ranging from fully transparent solar-cells, touch sensors, and OLEDs to transparent pixelated LEDs and micro-heaters based on a single layer of AgNWs. It is therefore believed that this method represents an important step toward the simple fabrication of high-performance flexible, biocompatible, and wearable devices.

## Methods

### Materials

Polyvinyl butyral (PVB, Butvar B-98, Mn = ~36,000 g/mol) with hydroxyl content in polyvinyl alcohol of approximately 18%, was purchased from Acros Organics. Hexamethylene diisocyanate (HDI) and N,N’-dimethylformamide (DMF) were obtained from Sigma-Aldrich Industry, USA. All chemicals were used as received without purification. A 0.5 wt% AgNWs solution dispersed in iso-propanol (IPA) was purchased from Nanopyxis, Korea. The average diameter and length of the nanowires were 35 nm and 20 μm, respectively.

### Intense Pulsed Light System

A custom-made intense pulsed light system was used for the photonic sintering and used a primary xenon flash lamp as the source (PerkinElmer QXF, UK). A water cooling system worked to maintain the temperature stability of the lamp, and a motion controller adjusted the z-axis translation stage for proper exposure of the fabricated films. The intense pulse light from the xenon flash lamp had a broad spectrum of light from 400 to 1000 nm. Sintering conditions were controlled by various factors such as voltage, pulse duration, pulse numbers, and operating time. The irradiation energy was measured by a NOVA II laser power meter (OPHIR), and the conductivity measurements were performed using a four-point probe method.

### OLED device fabrication

OLED Fabrication: The organic layers (small molecule) and cathode (Al) were evaporated using a thermal evaporator (10^−6^ Torr). The devices were fabricated with a 60-nm-thick N,N′-Bis(naphthalen-1-yl)- N,N′-bis(phenyl)-benzidine (NPB) layer as a hole transport layer, an 80-nm-thick tris(8-hydroxyquinolinato) aluminum (Alq_3_) layer as an electron transporting emissive layer, a 0.8-nm-thick lithium fluoride (LiF) layer as an electron injection layer, and a 100-nm-thick Al layer as a highly reflective cathode.

### Characterization

Surface morphologies and surface profiles were measured by field enhanced scanning electron microscopy (FE-SEM, S-4800, HITACHI Inc.) and atomic force microscopy (AFM, XE-100, Park system Inc.), respectively. The optical transmittances were measured with a UV-NIR spectrophotometer (Cary 5000, Agilent Technologies Inc.), and the EL characteristics were measured using a spectroradiometer (Spectra-Scan PR-670, Photo Research, Inc.) in a dark box (≈0.1 lx, at room temperature) with an adjustable voltage source meter (Model 237, Keithley Instruments, Inc.). The sheet resistance measurement was carried out by a standard four-point probe system (Resistivity meter, FPP-2400, Dasol ENG Inc.). A cyclic bending test was performed using a bending tester (Z-tec, Inc.) with a digital multimeter to measure the resistance change.

## Electronic supplementary material

Supplementary Information
