# Supplementary material for: Light sintering of ultra-smooth and robust silver nanowire networks embedded in poly(vinyl-butyral) for flexible OLED
Source: Sci Rep. 2018 Sep 21;8:14170. doi: 10.1038/s41598-018-32590-0 (PMC6155039; doi:10.1038/s41598-018-32590-0)
Supplement: Supplementary file 1 — Supplementary Information [file 41598_2018_32590_MOESM1_ESM.docx]

**Light sintering of ultra-smooth and robust silver nanowire networks embedded in poly(vinyl-butyral) for flexible OLED**

**(Supplementary Information)**

**Dong Jun Lee^1^,** [**Youngsu Oh**](http://pubs.rsc.org/en/results?searchtext=Author:Youngsu%20Oh)**^1,2^,**[**Jae-Min Hong**](http://pubs.rsc.org/en/results?searchtext=Author:Jae-Min%20Hong)**^3^, Young Wook Park^4,*^, and Byeong-Kwon Ju^1,*^**

^1^Display and Nanosystem Laboratory, College of Engineering, Korea University, Seoul 136-713, Republic of Korea

^2^ Photo-Electronic Hybrids Research Center, Korea Institute of Science and Technology (KIST), Seoul 02792, Republic of Korea

^3^ Institute of Advanced Composite Materials, Korea Institute of Science and Technology (KIST), Jeonbuk 55324, Republic of Korea

^4^ School of Mechanical and ICT Convergence Engineering, SUN MOON University, Chungcheongnam-do 31460, Republic of Korea

^*^Correspondence and requests for materials should be addressed to Y.W.P. (email: [zerook@sunmoon.ac.kr](mailto:zerook@sunmoon.ac.kr)) or B.-K.J. (email: bkju@korea.ac.kr)

Phone No.: +82-2-3290-3665

Fax. No.: +82-2-3290-3791

**Supplementary Information:**


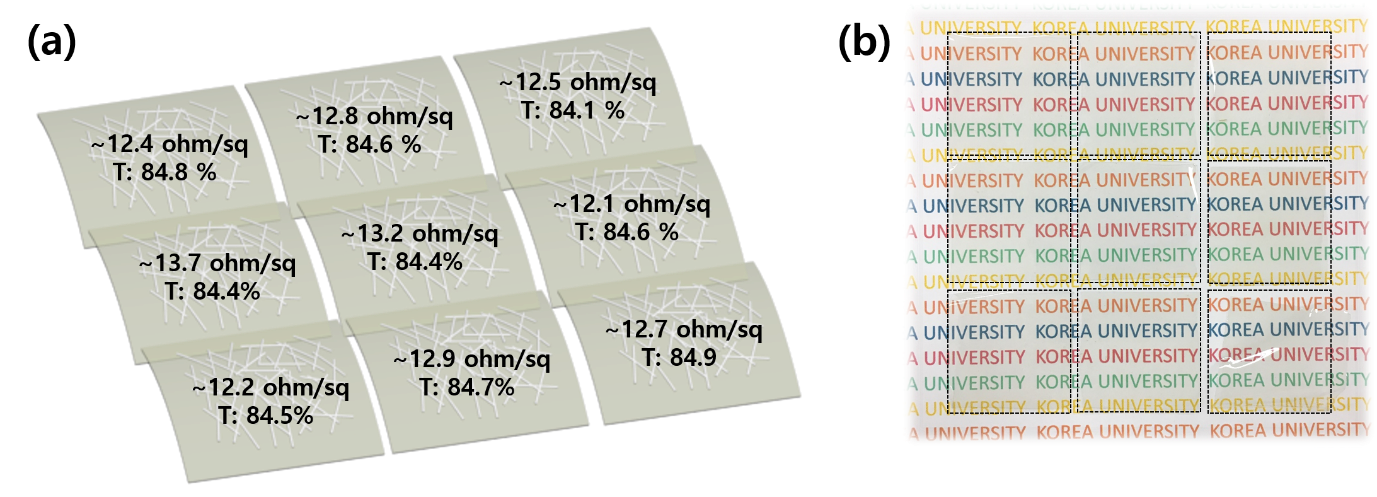


**Figure S1.** (a) Surface resistance and transmittance of large-area PAI electrode. (b) Actual photo of PAI electrode.

We fabricated 9×9 cm^2^ PVB/AgNWs with IPL(PAI) electrodes. In the nine sections, each side resistance and transmittance was measured. Figure S1 (a) shows the values of sheet resistance and transmittance for each section. The transmittance was measured based on a wavelength band of 550 nm, and the sheet resistance was measured at four places in each section and the average value was recorded. The measured transmittance was 84.5% on average and there were no significant changes among sections. In addition, the surface resistance was an average of 12.7 Ω/sq, and it was confirmed that there were no significant changes. Therefore, it has been confirmed that the PAI electrode has uniform electrical and optical characteristics. This information has been added to the supplementary section of the paper.

**Figure S2.** Haze spectrum of visible region of PVB / AgNW with IPL and PVB / AgNW without IPL.

**Figure S3.** Reflectance spectrum of IPL-treated and non-treated pristine PVB/AgNWs, and PET/IZO films.


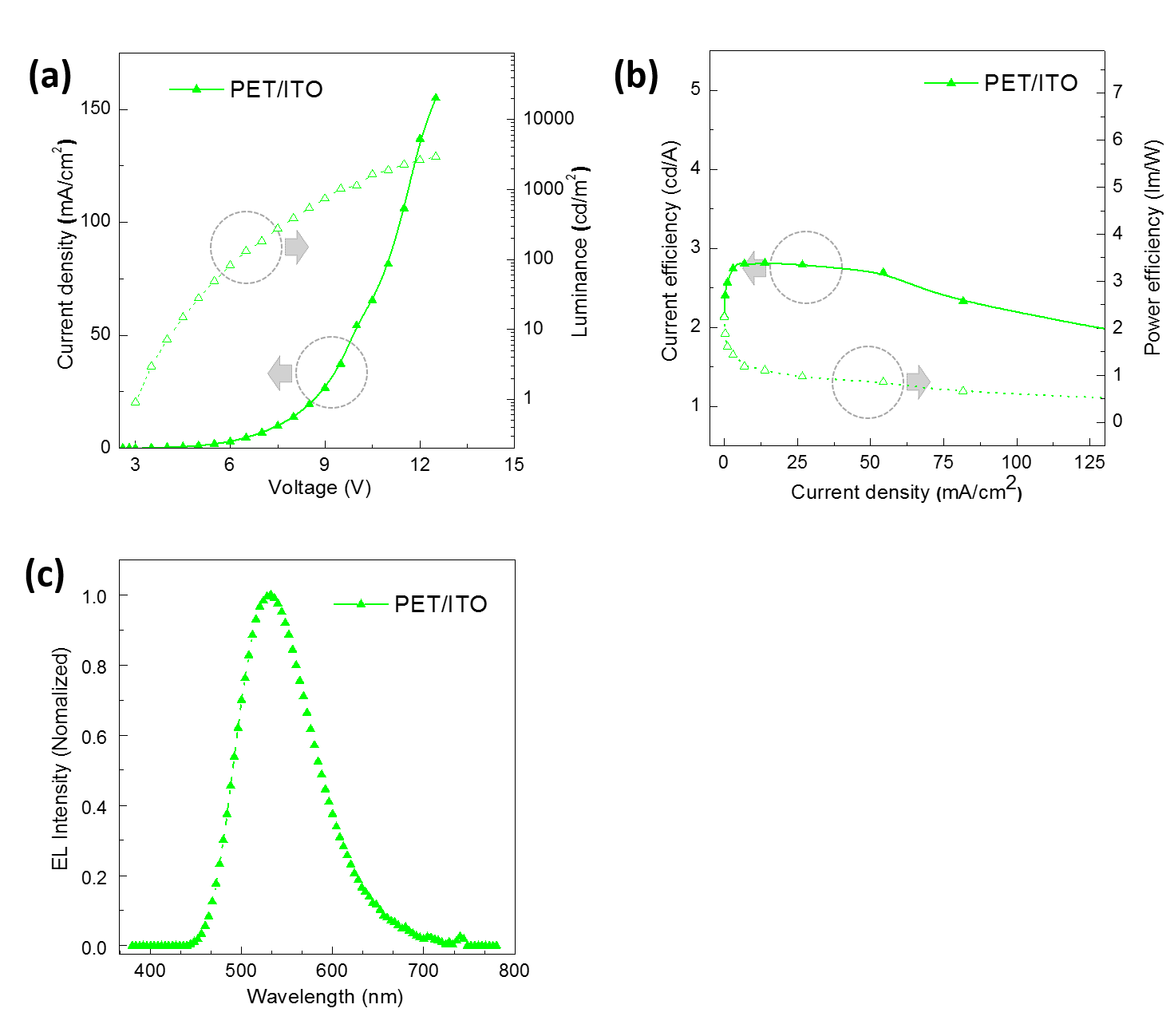


**Figure S4.** Characteristics of OLED devices with PET/ITO anode. (a) Current density–voltage-luminance characteristics. (b) Current efficiency and power efficiency as a function of luminance. (c) Normalized EL intensity versus wavelength.

| Device | Maximum CE^a)^  [cd/A] | Maximum PE^b)^  [lm/W] | Average ^c)^ | | Standard Deviation ^c)^ | |
| --- | --- | --- | --- | --- | --- | --- |
|  |  |  | CE [cd/A] | PE [lm/W] | CE [cd/A] | PE [lm/W] |
| PET/ITO | 2.81 | 2.24 | 2.32 | 0.67 | 0.34 | 0.78 |

^a)^ Maximum CE: maximum current efficiency; ^b)^ Maximum PE: maximum power efficiency; ^c)^ Current density from 25 mA/cm^2^ to 150 mA/cm^2^

**Table S1**. Device characteristics of OLEDs with PET/ITO electrodes.
